# A preliminary survey of antibiotic residues in frozen shrimp from retail stores in the United States^☆^

**DOI:** 10.1016/j.crfs.2021.09.009

**Published:** 2021-09-28

**Authors:** Robert P. Davis, D. Allen Davis, Claude E. Boyd

**Affiliations:** Auburn Univeristy, School of Fisheries, Aquaculture, and Aquatic Sciences, 203 Swingle Hall, Auburn, Al, 36849, United States

**Keywords:** Antibiotics, Shrimp, Sustainability, Food safety

## Abstract

Shrimp are an important and valuable commodity for aquaculture that are widely traded internationally. Widespread antibiotic use has been documented in shrimp farming and is a common source of criticism of aquaculture products. Additionally, previous reports have found some evidence of antibiotic residues in shrimp samples obtained from retail stores in the United States, which is a concern for consumers. To further understand the prevalence of antibiotics in retail shrimp in the United States, shrimp samples obtained from grocery stores across 16 states were analyzed for 74 antibiotic compounds/metabolites at a commercial laboratory. 68 samples were analyzed for a multiclass antibiotic panel which included 66 antibiotics while a subset of 15 samples were analyzed for β-lactam antibiotics, Nitrofurans, and Oxytetracycline. Samples were obtained that were labeled as being from major production countries, including India, Indonesia, Thailand, and Vietnam. No detectable antibiotic residues were found in this survey in any samples. This is contrary to previous findings in frozen shrimp analyzed for antibiotics, which typically report low levels of the prevalence of antibiotics.

## Highlights

• Shrimp were collected from grocery stores in various locations in the US.
• 68 samples were tested for a panel of 66 antibiotics.
• 15 samples were tested for β-lactam, nitrofuran, and tetracycline residues.
• Testing showed no positive results for antibiotic residues.
• Larger surveys are needed to determine the presence of antibiotics in shrimp products.

## Graphical abstract

**Figure:**
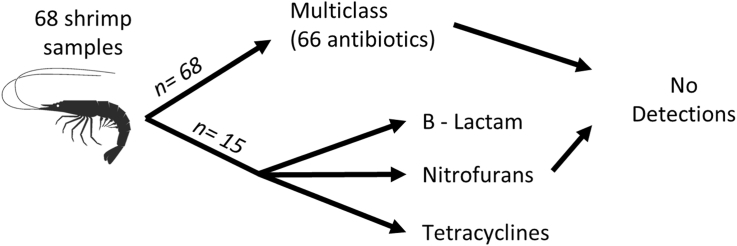

## 1 Introduction

The world's population is set to increase to 9 to 10 billion by 2050 and protein production will have to increase at a disproportionally higher rate due to the increase in standard of living, particularly in developing countries (FAO 2009). Seafood is an important part of the protein production in the world's food supply as it provides nutritious proteins along with Omega 3 fatty acids that are known for health benefits in humans (Boyd and McNevin 2014). Altogether, capture fisheries and aquaculture produce about 19% of the world's animal proteins for human consumption (Edwards et al., 2019). Fisheries production has plateaued over the last three decades, and aquaculture now accounts for about 50% of all seafood products (FAO 2018). Aquaculture is the only practical way to continue expanding seafood production.

**Fig. 1:**
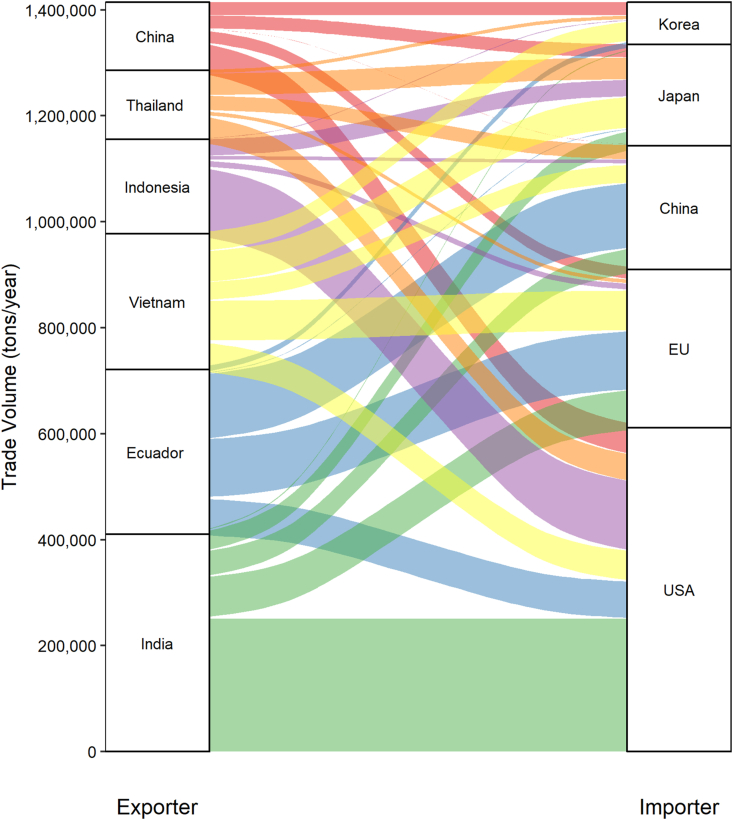
A Sankey diagram of trade flows from major shrimp exporters to major shrimp importers based on the United Nations COMTRADE Database for the year 2018. All product codes that include “shrimp” in the description were included, except for product codes reserved for coldwater shrimps (Pandalus spp.).

Shrimp are disproportionally valuable as an aquaculture species compared to their overall contribution to aquaculture production. Currently, there is about 6 million metric tons of Penaeid shrimp production globally, although a vast majority of this production is centered in Southeast Asia (FAO 2020). Shrimp is mainly produced as an export commodity, and the top markets in the world include the United States, European Union, Japan, Korea, and China (UN 2020). According to the United Nations commodities trading database (COMTRADE), the number one exporter of shrimp products is India, followed by Ecuador and several Southeast Asian countries that are also on the list of top producers (UN 2020, see Fig. 1).

One of the issues that shrimp producers face during the production process is disease outbreaks at the farm. In the past, this has manifested in the use of antibiotics, which is a common critique of shrimp aquaculture (e.g., Primavera 2005; Paul and Vogl 2011). Several studies have shown antibiotic residues in shrimps sampled from shrimp farms in Southeast Asia and Central America. For example, Swapna et al. (2012) found detectable levels of Chloramphenicol, Sulfonamide, Tetracycline, Erythromycin, Streptomycin, β-lactam antibiotics in shrimp farmed in major shrimp producing provinces in India. Holmstrom et al. (2003) found that 74% of farmers interviewed in Thailand in the year 2000 admitted to using antibiotics. Interestingly, surveys conducted by Boyd et al. (2017) and Boyd et al. (2018) in Thailand, Vietnam, and India reported zero farmers willing to disclose antibiotics use, which reflects an awareness that has arisen within shrimp farmers about the perceptions of antibiotics.

The inappropriate use of antibiotics at the farm level ultimately results in custom agencies denying entry of the product. Between 2002 and 2019, import refusals involving products that contained shrimp averaged 341 annually for the United States, and antibiotics were cited as a reason for refusal in over 1600 cases in the same period (annual average of 29% of total refusals, FDA 2020b). Interestingly, there has been a steady decline in the number of import refusals of shrimp products, while antibiotics have neither increased or decreased, statistically speaking. Within shrimp refusals, antibiotic drug residues were the second most common reason for refusal, behind a litany of refusal codes related to unsanitary/spoiled products. The prevalence with which antibiotic residues are in food for consumers remains a concern, especially since residues have been reported in retail samples in the past (Consumer Reports 2015; Done and Halden 2015; Tittlemier et al., 2007).

Therefore, the objective of this work was to understand the prevalence of antibiotics in shrimp from grocery retailers in the United States. The samples in this study were collected as part of another work based on elemental concentrations in shrimp tissue haphazardly, with no intention of targeting samples that may be positive for antibiotics. Antibiotic classes that were tested for were chosen in consultation with an aquatic animal health specialist, and because of their prevalence in reported failed import inspections (e.g., Chloramphenicol and Nitrofurans), importance to human health or agriculture (e.g., oxytetracycline compounds and β-Lactam compounds), and status as toxic agents in humans (e.g., Dimetridazole).

## 2 Methods

### 2.1 Sampling

**Fig. 2:**
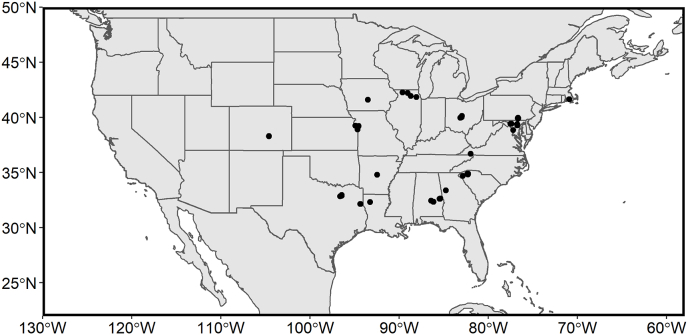
A map of the sampling locations of retail stores where shrimp were purchased for this study.

Shrimp were collected from stores in the US between January and August of 2019. Stores were selected to cover a broad range of stores that are owned by unique parent companies and sample from a range store types (e.g., high-end organic markets, budget markets, private membership clubs, regional store chains, and national store chains). In total, 57 stores were sampled in the United States, mostly in the South, East, and Midwest (Fig. 2). A complete list of stores sampled, and samples obtain is available in the supplementary information. At each location, bags of private label and store label frozen shrimp from the supermarket's freezer section were purchased, if available. A total of 68 samples were purchased, with samples being collected with the labeled country of origin as being Honduras (n = 1), India (n = 28), Indonesia (n = 25), Thailand (n = 11), and Vietnam (n = 3). At least 355 g (a 12-ounce bag) of shrimp for food brands (e.g., chicken of the sea) or store brands (e.g., Kroger branded) were purchased and kept frozen until processing for analysis. One bag of frozen shrimp purchased in each store, regardless of size (ranged from 335 g to 900 g), was considered a “sample”, and a sub-sample of at least 205 g from each bag were sent for analysis. Samples were sent on dry ice to the analytical laboratory in sealed plastic bags prior to analysis.

### 2.2 Analytical procedures

Samples were analyzed for antibiotic residue at an International Standards Organization (ISO) standard 17,025 (International Organization for Standardization 2017) certified commercial analytical laboratory accredited by a third-party certification body for the antibiotic tests that follow. A multi-class analytical procedure based on Adams et al. (2017) was conducted to analyze the presence of a suite of 66 antibiotic metabolites. Briefly, samples were homogenized and mixed with acetic acid and acetonitrile (1:4 mixture) and centrifuged. The top layer of the separated mixture was subsequently decanted and used for analysis. This method utilizes ion chromatography coupled with tandem mass spectrometry to detect analytes. All samples were analyzed for this broad-spectrum multi-class antibiotics test. A subset of 15 samples were also analyzed for Nitrofuran metabolites, β-Lactam antibiotics, and Oxytetracycline antibiotics due to the costs of the analysis. Samples chosen as the subset were somewhat evenly distributed between major production countries represented within the sample, with five samples from India, four from Indonesia, and three from Thailand and Vietnam each. Samples were analyzed for Nitrofurans via a method developed by the United States Food and Drug Administration (US FDA 2006). This method utilizes liquid chromatography tandem mass spectroscopy (LC-MS-MS) and utilizes a hydrochloric acid digestion. β-Lactam antibiotics were analyzed via a method developed by the US Department of Agriculture (USDA 2011). This method is also LC-MS-MS. Oxytetracycline compounds were analyzed via the method in Kijak et al. (1999), which is via LC-MS-MS. All the results are reported with the detection limit for each analyte as well.

## 3 Results

A total of 68 samples were collected for the analysis conducted in this report, across 16 states in the United States. A total of 57 stores were sampled, and 60% of the samples were store brand (i.e., Kroger branded products in stores owned by Kroger), while the remaining samples were third party brands (e.g., Chicken of the Sea). Not all grocery stores sold store branded products, thus private label brands were purchased in these stores. Of the samples collected, 43% were certified through the BAP certification scheme, (1/29 1 star, 16/29 2 star, 1/29 3 star, 10/29 4 star, 1/29 labeled “BAP Certified”). Only one sample labeled as Aquaculture Stewardship Council certified was found, and several other generic “sustainability” labels were found as well (e.g., “Third Party Verified”, “Responsibly Sourced”).

No detectable levels of antibiotics were found in any samples (Table 1, Table 2). All the samples tested were Pacific whiteleg shrimp *Litopenaeus vannamei*, except for one sample of black tiger shrimp *Penaeus monodon* with Vietnam as the labeled country of origin.

**Table 1:** The results of multiclass antibiotics testing panel on retail shrimps obtained from throughout the United States. The Country of Origin is the listed country of origin on the bag the shrimp were purchased in. LOD = the limit of detection (ng/g), nd = below detection limits. The number of samples is listed next to the country in parentheses. Results are reported on as-is basis (i.e., wet weights).

Antibiotic	LOD (ng/g)	Labeled Country of Origin				
		Honduras (1)	India (28)	Indonesia (25)	Thailand (11)	Vietnam (3)
Cefalexin	5	n.d.	n.d.	n.d.	n.d.	n.d.
Cefapirin	10	n.d.	n.d.	n.d.	n.d.	n.d.
Cefazolin	50	n.d.	n.d.	n.d.	n.d.	n.d.
Cefaperazon	50	n.d.	n.d.	n.d.	n.d.	n.d.
Cefquinome	10	n.d.	n.d.	n.d.	n.d.	n.d.
Ceftiofur	100	n.d.	n.d.	n.d.	n.d.	n.d.
Cephalonium	10	n.d.	n.d.	n.d.	n.d.	n.d.
Ciprofloxacin	10	n.d.	n.d.	n.d.	n.d.	n.d.
Danofloxacin	10	n.d.	n.d.	n.d.	n.d.	n.d.
Difloxacin	10	n.d.	n.d.	n.d.	n.d.	n.d.
Enoxacin	10	n.d.	n.d.	n.d.	n.d.	n.d.
Enrofloxacin	10	n.d.	n.d.	n.d.	n.d.	n.d.
Erythromycin	50	n.d.	n.d.	n.d.	n.d.	n.d.
Lincomycin	50	n.d.	n.d.	n.d.	n.d.	n.d.
Lomefloxacin	10	n.d.	n.d.	n.d.	n.d.	n.d.
Marbofloxacin	10	n.d.	n.d.	n.d.	n.d.	n.d.
Nalidixic Acid	50	n.d.	n.d.	n.d.	n.d.	n.d.
Norfloxacin	10	n.d.	n.d.	n.d.	n.d.	n.d.
Ofloxacin	10	n.d.	n.d.	n.d.	n.d.	n.d.
Oleandomycin	50	n.d.	n.d.	n.d.	n.d.	n.d.
Oxolinic acid	10	n.d.	n.d.	n.d.	n.d.	n.d.
Sarafloxacin	10	n.d.	n.d.	n.d.	n.d.	n.d.
Spiramycin	50	n.d.	n.d.	n.d.	n.d.	n.d.
Sulfabenzamide	10	n.d.	n.d.	n.d.	n.d.	n.d.
Sulfachloropyridazine	10	n.d.	n.d.	n.d.	n.d.	n.d.
Sulfaclozine (Sulfaclorpryazine)	10	n.d.	n.d.	n.d.	n.d.	n.d.
Sulfadiazine	10	n.d.	n.d.	n.d.	n.d.	n.d.
Sulfadimethoxine	10	n.d.	n.d.	n.d.	n.d.	n.d.
Sulfadoxine	10	n.d.	n.d.	n.d.	n.d.	n.d.
Sulfamerazine	10	n.d.	n.d.	n.d.	n.d.	n.d.
Sulfamethazine	10	n.d.	n.d.	n.d.	n.d.	n.d.
Sulfamethizole	10	n.d.	n.d.	n.d.	n.d.	n.d.
Sulfamethoxazole	10	n.d.	n.d.	n.d.	n.d.	n.d.
Sulfamoxole	10	n.d.	n.d.	n.d.	n.d.	n.d.
Sulfapyridine	10	n.d.	n.d.	n.d.	n.d.	n.d.
Sulfaquinoxaline	10	n.d.	n.d.	n.d.	n.d.	n.d.
Sulfathiazole	10	n.d.	n.d.	n.d.	n.d.	n.d.
Sulfisoxazole	10	n.d.	n.d.	n.d.	n.d.	n.d.
Tilmicosin	50	n.d.	n.d.	n.d.	n.d.	n.d.
Trimethoprim	10	n.d.	n.d.	n.d.	n.d.	n.d.
Tylosin	50	n.d.	n.d.	n.d.	n.d.	n.d.
Virginiamycin M1	50	n.d.	n.d.	n.d.	n.d.	n.d.
Cambendazole	5	n.d.	n.d.	n.d.	n.d.	n.d.
Chloramphenicol (CAP)	5	n.d.	n.d.	n.d.	n.d.	n.d.
Cloxacillin	5	n.d.	n.d.	n.d.	n.d.	n.d.
Dicloxacillin	5	n.d.	n.d.	n.d.	n.d.	n.d.
Dimetridazole (DMZ)	5	n.d.	n.d.	n.d.	n.d.	n.d.
Fenbendazole	50	n.d.	n.d.	n.d.	n.d.	n.d.
Ipronidazole (IPZ)	5	n.d.	n.d.	n.d.	n.d.	n.d.
Ipronidazole metabolite (IPZ-OH)	5	n.d.	n.d.	n.d.	n.d.	n.d.
Mebendazole	5	n.d.	n.d.	n.d.	n.d.	n.d.
Metronidazole (MNZ)	5	n.d.	n.d.	n.d.	n.d.	n.d.
Nafcillne	5	n.d.	n.d.	n.d.	n.d.	n.d.
Oxacillin	5	n.d.	n.d.	n.d.	n.d.	n.d.
Oxfendazole	5	n.d.	n.d.	n.d.	n.d.	n.d.
Penicillin G	5	n.d.	n.d.	n.d.	n.d.	n.d.
Ronidazole	5	n.d.	n.d.	n.d.	n.d.	n.d.
Thiabendazole	5	n.d.	n.d.	n.d.	n.d.	n.d.
Thiamphenicol	5	n.d.	n.d.	n.d.	n.d.	n.d.

**Table 2:** The results of antibiotics testing on a subset of samples obtained from throughout the United States tested for β-Lactam antibiotics, nitrofurans, and oxytetracycline. The country of origin is the listed country of origin on the bag the shrimp were purchased in. LOD = the limit of detection (ng/g), nd = below detection limits. The number of samples is listed next to the country in parentheses. Results are reported on as-is basis (i.e., wet weights).

Antibiotic	LOD (ng/g)	Labeled Country of Origin			
		India (5)	Indonesia (4)	Thailand (3)	Vietnam (3)
**β - Lactam Antibiotics**					
Amoxicillin	2	n.d.	n.d.	n.d.	n.d.
Ampicillin	3	n.d.	n.d.	n.d.	n.d.
Cloxacillin	4	n.d.	n.d.	n.d.	n.d.
Dicloxacillin	5	n.d.	n.d.	n.d.	n.d.
Oxacillin	3	n.d.	n.d.	n.d.	n.d.
Penicillin G	4	n.d.	n.d.	n.d.	n.d.
Nafcillin	5	n.d.	n.d.	n.d.	n.d.
**Nitrofurans**					
AOZ (3-amino-2-oxazolidinone)	1	n.d.	n.d.	n.d.	n.d.
AMOZ (5-Methylmorpholino-3-amino-2-oxazolidinone)	1	n.d.	n.d.	n.d.	n.d.
SC (Semicarbazide)	1	n.d.	n.d.	n.d.	n.d.
AH (1-Aminohydantoin)	1	n.d.	n.d.	n.d.	n.d.
**Tetracyclines**					
Oxytetracycline	10	n.d.	n.d.	n.d.	n.d.
Tetracycline	10	n.d.	n.d.	n.d.	n.d.
Chlortetracycline	10	n.d.	n.d.	n.d.	n.d.
Doxycycline	20	n.d.	n.d.	n.d.	n.d.

## 4 Discussion

Penaeid shrimp aquaculture accounted for 6 million metric tons of production of shrimp in 2018, with over 5 million metric tons accounted for by *Penaeus monodon* and *Litopenaeus vannamei* (FAO 2020). Crustaceans and shrimp are disproportionally valuable as an aquaculture commodity, and shrimp are grown mainly for export, with the exception of a few countries (e.g., China) where domestic consumption is high. Shrimp aquaculture began as a means to supplement lagging fisheries harvests and has since grown to meet rising demands. The use of antibiotics in shrimp farming is well known and previously documented (Holmstrom et al., 2003; Smith 2010), and a common critique of shrimp farming from environmental groups (e.g., Ma 2015). Additionally, antibiotic residues have been documented in samples of shrimp purchased in retail stores and during customs checks, which means that it is a concern for consumers and public health. This work was aimed at determining the prevalence of antibiotics in shrimp purchased from retail stores in the United States between January and August 2019.

Detectable levels of antibiotics have been previously identified in shrimp samples taken from retail stores. Johnson et al. (2014) found detectable levels of malachite green, chloramphenicols, nitrofurans, and fluoroquinones, with 93% of all samples testing positive for at least one analyte. Other studies on retail samples have reported fewer positive cases. Done et al. (2015) found that farmed shrimp tested positive for only one analyte out of 47 antibiotic analytes tested (sulfadimethoxine). Tittlemeier et al. found 5 out of 39 analytes in homogenized shrimp tissue samples across a decade of sampling (Quinolones and Nitrofurans), at low concentrations. Khan and Lively (2020) reported positive tests for fluoroquinolone and oxytetracycline shrimp purchased in Louisiana with a novel analytical procedure, but not for other analytes. This survey found no detectable antibiotics from retail stores, which suggests that there may be patchiness to possible contamination across distribution networks and time. While the limits of detection in these other studies were lower than the present study, the limit of detection for the antibiotics analyzed in this study were below or on the same order of magnitude as the regulatory action level for the FDA in nearly all cases (FDA, 2020a), and previous efforts in broad antibiotic screening in seafood (Done and Halden, 2015). This suggests that current testing was at an appropriate detection level, and missed detections were not likely a result of poor analytic resolution.

**Fig. 3:**
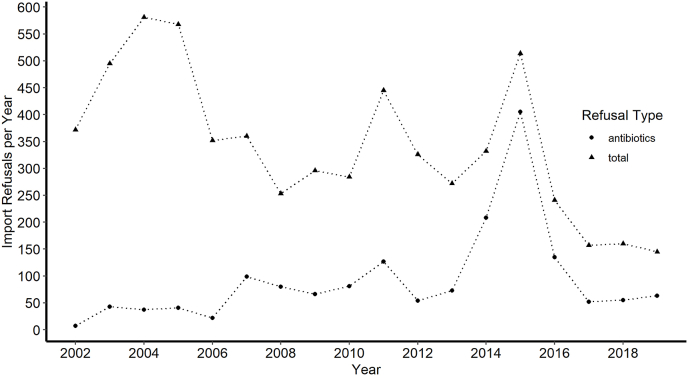
The number of annual import refusals for shrimp products (any product with “shrimp” in the description) in the US import refusal database through 2019, the year of the sampling in this study. Circles = all refusals, while triangles = the number of refusals with antibiotics listed as a reason for refusal.

Antibiotic residues in imported seafood are likely to continue to be a concern for consumers for the foreseeable future. Fig. 3 shows the trend in recent years with regards to import refusals of shrimp products. As shown, antibiotic residue is a driver of shrimp refusals, and continues to be an issue for shrimp products. Evidence from field surveys suggests that aquaculture is becoming more intensive as demand increases (Boyd et al. 2017, 2018). Unfortunately, only a small fraction of imports are tested by the FDA (GAO 2017), and therefore the potential for shipments with antibiotic tainted shrimp to slip through the cracks remains. Contrarily to previous studies, no antibiotics were found in the samples in this study, although another recent study with farmed shrimp found little evidence of antibiotic contamination as well (Done and Halden 2015). However, a growing concern in seafood products are antibacterial resistant microbes, which have been documented recently in frozen seafood products (Thornber et al., 2020; Chajecka-Wierzchowska et al., 2016). This study did not explore microbial contamination but given the prevalence of microbial contamination and spoiled products in import refusals, future efforts would be strengthened by coupling the exploration of both antibiotic residues and microbial contamination. Finally, future research efforts should include spot sampling of retail samples to understand the prevalence of antibiotic residues in food purchased for consumption. The authors.

## CRediT authorship contribution statement

**Robert P. Davis:** Formal analysis, Data curation, Investigation, Conceptualization, Methodology, Visualization, Writing – original draft, Writing – review & editing. **D. Allen Davis:** Funding acquisition, Investigation, Conceptualization, Methodology, Data curation, Formal analysis, Writing – review & editing. **Claude E. Boyd:** Funding acquisition, Investigation, Conceptualization, Methodology, Data curation, Formal analysis, Writing – review & editing.

## Declaration of competing interest

The authors declare that they have no known competing financial interests or personal relationships that could have appeared to influence the work reported in this paper.
