# Supplementary material for: A preliminary survey of antibiotic residues in frozen shrimp from retail stores in the United States
Source: Curr Res Food Sci. 2021 Sep 28;4:679–83. doi: 10.1016/j.crfs.2021.09.009 (PMC8495020; doi:10.1016/j.crfs.2021.09.009)
Supplement: Multimedia component 1 [file mmc1.docx]

Supplementary Information. A table of samples included in this study. Bolded rows are sampled that were selected for β-Lactam, Nitrofuran, and Tetracycline testing (n=15).

| ID no. | Store | State Sampled | Brand | Labeled Country of Origin | Size | Certification Status |
| --- | --- | --- | --- | --- | --- | --- |
| 1 | Weis | Pennsylvania | Weis Organic | Indonesia | 31-40 CT | No Certification |
| 2 | Weis | Pennsylvania | Weis Quality | Indonesia | 16-20 CT | No Certification |
| 3 | Giant | Pennsylvania | Nature Promise (Giant Organic) | India | 31-40 CT | No Certification |
| **4** | **Giant** | **Pennsylvania** | **Giant Brand** | **Thailand** | **51-60 CT** | **No Certification** |
| **5** | **Save-a-Lot** | **Pennsylvania** | **Captain Bob's** | **India** | **40-60 CT** | **HACCP** |
| **6** | **Sam's Club** | **Pennsylvania** | **Membersmark** | **India** | **50-70 CT** | **BAP 2 Star** |
| **7** | **Nell's Market** | **Pennsylvania** | **Wholey** | **Vietnam** | **26-30 CT** | **HACCP** |
| 8 | Nell's Market | Pennsylvania | Best Yet | Indonesia | 31-40 CT | HACCP |
| 9 | Price Rite | Pennsylvania | Tastee Choice | India | 31-40 CT | BAP 2 Star |
| **10** | **Price Rite** | **Pennsylvania** | **Cape Gourmet** | **Indonesia** | **31-40 CT** | **BAP 2 Star** |
| 11 | Grocery Outlet | Pennsylvania | Ocean Gift | Indonesia | 26-30 CT | BAP 2 Star |
| 12 | Grocery Outlet | Pennsylvania | Chicken of the Sea | Indonesia | 16-20 CT | BAP 1 Star |
| **13** | **Grocery Outlet** | **Pennsylvania** | **CenSea** | **Indonesia** | **31-40 CT** | **No Certification** |
| **14** | **Wegmans** | **Maryland** | **Ultra** | **Thailand** | **21-25 CT** | **BAP 3 Star** |
| **15** | **BJ's** | **Maryland** | **Wellsley Farms** | **India** | **21-25 CT** | **No Certification** |
| 16 | Safeway | Maryland | Waterfront Bistro | Indonesia | 31-40 CT | No Certification |
| 17 | Giant Eagle | Maryland | Sail | Indonesia | 26-30 CT | No Certification |
| 18 | Giant Eagle | Maryland | SeaMazz | Indonesia | 31-40 CT | BAP 2 Star |
| 19 | Giant Eagle | Maryland | Nature's Basket | India | 26-30 CT | No Certification |
| 20 | Moms Organic Market | Virginia | BMR | India | 16-20 CT | No Certification |
| 21 | Food City | Virginia | Cape Covell | Indonesia | 41-50 CT | No Certification |
| 22 | Lowes Foods | South Carolina | Black Tie | Thailand | 26-30 CT | HACCP, BAP 1 Star, "Turtle Safe" |
| **23** | **Bi Lo** | **South Carolina** | **Fishermans Wharf** | **Indonesia** | **16-20 CT** | **BAP 4 Star** |
| 24 | Ingles | South Carolina | Seabest | India | 26-30 CT | BAP 2 Star |
| 25 | Lidl | South Carolina | No Branding (Store Brand) | Thailand | 31-40 CT | BAP 2 Star |
| 26 | Trader Joes | South Carolina | Trader Joes | Thailand | 21-30 CT | No Certification |
| 27 | Aldi | Alabama | Fremont Fish Market | India | 41-60 CT | BAP 4 Star |
| **28^1^** | **Whole Foods** | **Alabama** | **"Farmed Raised Shrimp"** | **Vietnam** | **51-60 CT** | **"Third Party Cert** |
| **29** | **Costco** | **Alabama** | **Kirklands** | **India** | **50-70 CT** | **No Certification** |
| 30 | Target | Alabama | Market Pantry | Indonesia | 41-50 CT | BAP 2 Star |
| 31 | Kroger | Alabama | Aquastar | Indonesia | 50 Shrimp | "Responsibily Sourced' |
| 32 | Kroger | Alabama | Kroger | Indonesia | Large | BAP 2 Star |
| 33 | Piggly Wiggly | Alabama | Natures Best | India | 41-50 CT | No Certification |
| 34 | Walmart | Alabama | Walmart Brand | India | 41-60 CT | BAP 4 Star |
| **35** | **Walmart** | **Alabama** | **Captains Pack** | **India** | **31-40 CT** | **BAP 4 Star** |
| 36 | Publix | Alabama | Publix | Indonesia | 31-35 CT | No Certification |
| 37 | Publix | Alabama | Chicken of the Sea | Thailand | 13-15 CT | BAP 2 Star |
| 38 | Natural Grocers | Colorado | SeaJoy | Honduras | 36-40 CT | BAP 4 Star and ASC |
| 39 | Albertsons | Colorado | Waterfront Bistro | Indonesia | 31-40 CT | No Certification |
| 40 | Albertsons | Colorado | Waterfront Bistro | Indonesia | 26-30 CT | No Certification |
| 41 | Schnucks | Illinois | KNC Agro Lmtd | India | 16-20 CT | None |
| 42 | Hyvee | Illinois | Hyvee Fish Market | Thailand | 26-30 CT | BAP 2 Star |
| 43 | Target | Kansas | Market Pantry | India | 41-50 CT | BAP 2 Star |
| **44** | **Jewel-Osco** | **Illinois** | **Waterfront Bistro** | **Indonesia** | **26-30 CT** | **None** |
| **45** | **Hyvee** | **Missouri** | **Hyvee Fish Market** | **Thailand** | **26-30 CT** | **BAP 2 Star** |
| 46 | Tom Thumb | Texas | Waterfront Bistro | Indonesia | 26-30 CT | No Certification |
| 47 | Costco | Texas | Kirklands | India | 21-25 CT | No Certification |
| 48 | Fareway Foods | Iowa | Supreme Choice | Indonesia | 31-40 CT | None |
| 49 | Dillons | Kansas | Kroger | India | extra large' | No Certification |
| 50 | Cubs | Illinois | Artic Shores | Indonesia | 26-30 CT | BAP 2 Star |
| **51** | **Winco** | **Texas** | **Winco** | **Vietnam** | **41-50 CT** | **BAP 4 Star** |
| 52 | Price Chopper | Missouri | Aquastar | Indonesia | 31-40 CT | BAP 2 Star |
| 53 | Walmart | Arkansas | Walmart Brand | India | 26-30 CT | BAP 4 Star |
| 54 | Brookshire Brothers | Texas | Brookshire Brothers | India | 51-60 CT | No Certification |
| 55 | Brookshire | Lousiana | Full Cirlce | India | 21-25 CT | No Certification |
| 56 | Supervalu | Lousiana | Diamond Reef | India | 31-40 CT | No Certification |
| 57 | Aldi | Texas | Aldi Fremont Market | India | 41-60 CT | BAP 4 Star |
| 58 | H-E-B | Texas | Great Catch | Indonesia | 26-30 CT | No Certification |
| 59 | Publix | Georgia | Publix | India | 41-50 CT | No Certification |
| 60 | Giant Eagle | Ohio | Giant Eagle | India | 26-30 CT | BAP 4 Star |
| 61 | Food Lion | South Carolina | Food Lion | India | 31-40 CT | BAP 2 Star |
| 62 | Lucky's | Ohio | Lucky Brand | India | 26-30 CT | No Certification |
| 63 | Food Lion | South Carolina | Oishi | Thailand | 13-15 CT | No Certification |
| 65 | Kroger | Ohio | Simple Truth | Thailand | "Jumbo" | No Certification |
| 66 | Meijer | Ohio | Meijer Brand | India | 26-30 CT | BAP 4 Star |
| 67 | Costco | Ohio | Kirklands | India | 31-40 CT | No Certification |
| 68 | HMart | Maryland | Tong Tong Bay | Thailand | 26-30 CT | No Certification |
| 69 | HMart | Maryland | Fresh | Indonesia | 26-30 CT | No Certification |

^1^This sample was black tiger shrimp (*Penaeus monodon*), while all other samples were whiteleg shrimp (*Litopenaeus vannamei*)
